# Does Parenteral Nutrition Influence Electrolyte and Fluid Balance in Preterm Infants in the First Days after Birth?

**DOI:** 10.1371/journal.pone.0009033

**Published:** 2010-02-03

**Authors:** Liset E. Elstgeest, Shirley E. Martens, Enrico Lopriore, Frans J. Walther, Arjan B. te Pas

**Affiliations:** Division of Neonatology, Department of Pediatrics, Leiden University Medical Center, Leiden, The Netherlands; University of Giessen Lung Center, Germany

## Abstract

**Background:**

New national guidelines recommend more restricted fluid intake and early initiation of total parenteral nutrition (TPN) in very preterm infants. The aim was study the effect of these guidelines on serum sodium and potassium levels and fluid balance in the first three days after birth.

**Methods:**

Two cohorts of infants <28 weeks gestational age, born at the Leiden University Medical Center in the Netherlands, were compared retrospectively before (2002–2004, late-TPN) and after (2006–2007, early-TPN) introduction of the new Dutch guideline. Outcome measures were serum sodium and potassium levels, diuresis, and changes in body weight in the first three postnatal days.

**Results:**

In the first three postnatal days no differences between late-TPN (N = 70) and early-TPN cohort (N = 73) in mean (SD) serum sodium (141.1 (3.8) vs 141.0 (3.7) mmol/l) or potassium (4.3 (0.5) vs 4.3 (0.5) mmol/l) were found, but in the early-TPN cohort diuresis (4.5 (1.6) vs 3.2 (1.4) ml/kg/h) and loss of body weight were decreased (−6.0% (7.7) vs −0.8% (8.0)).

**Conclusions:**

Initiation of TPN immediately after birth and restricted fluid intake in very preterm infants do not seem to influence serum sodium and potassium levels in first three postnatal days. Further research is needed to see if a decreased diuresis and loss of body weight in the first days is the result of a delayed postnatal adaptation or better energy balance.

## Introduction

During the first days of life very preterm infants are almost entirely dependent on total parenteral nutrition (TPN) to meet their energy and nutritional requirements.[1] Soon after birth preterm infants are at risk for catabolism. Early administration of TPN, including amino acids, directly after birth has beneficial effects on their nitrogen balance, neonatal growth, and health.[1]–[3] Intake of fluid and electrolytes is accomplished by TPN as well. Adequate management of fluid and electrolytes is essential in very preterm infants to prevent morbidity and mortality.[4], [5]

In the first postnatal days disturbances in the fluid and electrolyte balance occur frequently in very preterm infants as a result of high insensible water loss and renal immaturity.[6]–[9] This imbalance can lead to major complications, such as neurological impairment and cardiac arrhythmia caused by hypernatraemia and hyperkalaemia respectively.[4], [9]–[11] Other complications can result from a delayed loss of interstitial fluid from the extracellular fluid compartment, clinically indicated by a delayed postnatal weight loss.[4], [6], [12] Persistent expansion of the extracellular fluid compartment and retention of interstitial fluid are associated with an increased risk of respiratory morbidity [13]–[16], patent ductus arteriosus [17]–[19], and necrotising enterocolitis.[19], [20]

Although it is difficult to ascertain requirements of fluid and electrolytes, some general recommendations have been made. Intake of fluid should be restricted.[7], [8], [19] Administration of sodium should be started after the onset of postnatal diuresis and natriuresis from the second or third day after birth, or when weight loss of at least 6% of birth weight has been achieved.[1], [5]–[8], [21], [22] It is also recommended to delay supplementation of potassium until diuresis has started and renal function is clearly established.[1], [6], [8]

In 2005 the fluid and nutrition guidelines at our neonatal center have been changed. The changes were based on recent national guidelines.[23] Major modifications in the new regimen were (1) initiation of TPN, including sodium and potassium, immediately after birth and (2) restriction of fluid intake.

We performed a retrospective study to investigate whether the changes in fluid and nutrition policy had effect on (1) serum sodium and potassium levels and on (2) diuresis and changes in body weight in the first days after birth.

## Methods

All inborn infants less than 28 weeks' gestation, admitted to the neonatal intensive care unit of the Leiden University Medical Center between 1 January 2002 and 31 December 2004 (late TPN cohort), and between 1 January 2006 and 31 December 2007 (early TPN cohort), were retrospectively identified. Infants who died within 72 hours after birth or with severe congenital anomalies were excluded. Infants born in 2005 were not included to prevent a bias due to an adjustment period after the introduction of the new national fluid and nutrition guideline.

### Fluid and Nutrition Regimens

Intake of fluid, sodium, and potassium in the first 72 hours after birth for the former and new guideline are shown in table 1.

**Table 1:** Fluid and nutrition guidelines for the late and early TPN cohort.

FORMER GUIDELINE 2002, 2003, 2004		Day 1	Day 2	**START TPN**	Day 3
**Birth weight (gr)**		0–24 h	24–36 h	36–48 h	48–72 h
**<750**	Fluid intake	120	140		160
	Sodium intake	0	0	1.44	3.25
	Potassium intake	0	0	0.40	1.11
**750–1000**	Fluid intake	100	120		140
	Sodium intake	0	0	1.15	2.67
	Potassium intake	0	0	0.34	0.99
**1000–1250**	Fluid intake	80	100		120
	Sodium intake	0	0	1.00	2.38
	Potassium intake	0	0	0.31	0.93
NEW GUIDELINE 2006, 2007		Day 1 **START TPN**	Day 2		Day 3
**Birth weight (gr)**		0–24 h	24–48 h		48–72 h
**<1000**	Fluid intake	80	100		120
	Sodium intake	1.80	2.15		2.57
	Potassium intake	0.81	1.31		1.25
**1000–1500**	Fluid intake	60	80		100
	Sodium intake	1.22	1.66		1.99
	Potassium intake	0.69	0.94		1.13

Fluid intake is in mL/kg, Sodium and Potassium is in mmol/kg.

In the late TPN cohort fluid administration was started at 80–120 ml/kg/day and increased by 20 ml/kg/day to a maximum of 160 ml/kg/day. Preterm infants received glucose with calcium intravenously for approximately the first 36 hours after birth. Thereafter, depending on the infants' condition, and renal and liver functions, infusion of glucose with minerals was started or TPN, including glucose, amino acids, lipids, and minerals. When glucose with minerals was started, this was as soon as possible replaced by TPN. Glucose with minerals and the amino acid infusion of TPN both contained sodium and potassium.

In the early TPN cohort fluid administration started at 60–80 ml/kg/day increasing by 20 ml/kg/day to a maximum of 150 ml/kg/day. Furthermore, TPN was started as soon as possible after birth, resulting in an administration of sodium and potassium generally within the first postnatal hour.

In both cohorts minimal enteral feeding was started in the first day after birth.

### Data Collection

The following demographical and clinical characteristics were collected from medical records: gender, gestational age, birth weight, intrauterine growth retardation (IUGR, birth weight <3rd percentile), (use of) antenatal steroidş multiple gestation, and Apgar score at 5 minutes. From the first three days after birth data were collected on use of antibiotics, placement of umbilical arterial lines, volume expansion with normal saline (NaCl 0.9%), albumin, fresh frozen plasma, or ***pasteurised plasma protein*** solution, sodium bicarbonate administration, and erythrocyte transfusions. CRIB (Clinical Risk Index for Babies) scores were calculated for each infant by adding up the individual scores of six parameters (birth weight, gestational age, congenital malformations, maximum base excess in the first 12 hours after birth, and minimum and maximum fraction of inspired oxygen in the first 12 hours after birth).[24]

Outcome variables included serum sodium and potassium levels in the first three days of life. Serum concentrations of both electrolytes were noted starting from 6 hours after birth and noted every 6 hours, resulting in 12 measurements in the first 72 hours after birth. We defined hypernatraemia, hyponatraemia, hyperkalaemia, and hypokalaemia as respectively: serum sodium ≥150 mmol/l, serum sodium ≤130 mmol/l, serum potassium ≥6.5 mmol/l, and serum potassium ≤3.5 mmol/l, at least once during the first three days of life.

Data were collected on urine output in the first three days after birth and body weight on postnatal days 1, 2, 3, 14, and 21. Changes in body weight as percentage of birth weight were calculated. Other clinical outcomes recorded included: need and duration of mechanical ventilation, surfactant treatment, patent ductus arteriosus, necrotising enterocolitis, intraventricular hemorrhage, cystic periventricular leucomalacia, and retinopathy of prematurity. Most preterm infants are already transferred to regional district hospitals with special care units before they have reached the postnatal age of 36 weeks. We did not record the incidence of bronchopulmonary dysplasia as different policies of respiratory support and oxygen therapy would lead to a major bias in comparing the incidence between both cohorts.

The actual total daily fluid intakes during the first three postnatal days were noted and compared with those prescribed in the guidelines.

In the study period there were no other major changes in antenatal care, regimens, and protocols that could influence the electrolyte or fluid balance in the first three days after birth.

### Statistical Analyses

Data are presented as mean and standard deviation (SD) for normally skewed continuous variables or as median and interquartile range (IQR) for skewed variables, and as numbers and proportions (%) for categorical variables. All comparisons between the late and early TPN cohort were performed by using independent Student's *t* test for parametric and the Mann-Whitney *U* test for non-parametric continuous variables, and χ^2^-test or Fisher's exact test for categorical variables. Multivariable regression analysis was performed to adjust for possible confounding effects of additional sodium sources. Results of logistic models are presented as an odds ratio (OR) with 95% confidence interval (CI). Reported *P*-values are based on two-sided testing, and *P*-values <0.05 were considered statistically significant. All statistical analyses were carried out using SPSS (SPSS for Windows, version 16.0, 2008, Chicago, IL).

#### Ethics and consent

Due to the retrospective character of this study, approval of the Research Ethics Committee in our hospital was not needed. According to Dutch legislation written parental consent was not needed for this retrospective study. Data collected from medical records were stored with study numbers, but not with patient identifiers. The list of study numbers linked with patient identifiers are kept in a locked filing cabinet.

## Results

During both study periods a total of 166 (late TPN cohort vs early TPN cohort: 83 vs 83) inborn infants with gestational age <28 weeks were admitted to the neonatal unit. Twenty-three infants were excluded: 20 infants (11 vs 9) died within 72 hours after birth, one infant (in the early TPN cohort) was excluded because of severe fetal hydrops, and two infants (in the late TPN cohort) had incomplete medical records. The 20 infants who died within 72 hours had electrolyte levels within the normal range. Data of 143 infants were analysed.

There were no significant differences in demographic and clinical characteristics between both cohorts (table 2).

**Table 2:** Demographic and clinical characteristics of the late and early TPN cohort.

Characteristic	Late TPN	Early TPN	*P*-Value
	*n = 70*	*n = 73*	
Male gender, n (%)	38 (54)	45 (62)	0.4
Gestational age (weeks), median (IQR)	26 (26–27)	26 (26–27)	0.4
Birth weight (gr), mean (SD)	907 (172)	915 (171)	0.8
IUGR, n (%)	2 (3)	3 (4)	1.00*
Antenatal steroids, n(%)	55 (78)	59 (81)	0.7
Singletons, n (%)	38 (54)	50 (68)	0.1
Congenital malformations, n (%)	3 (4)	2 (3)	0.7
5-min Apgar score, median (IQR)	8 (7–9)	8 (6–9)	0.4
CRIB score, median (IQR)	4 (2–8)	3 (2–6)	0.3
Antibiotics in first 72 h, n (%)	69 (99)	72 (99)	1.0*
Arterial line, n (%)	62 (89)	60 (82)	0.3
Normal saline infusion in first 72 h (ml), median (IQR)	10 (7–20)	9 (0–20)	0.1
Albumin, ***pasteurised plasma protein***, and fresh frozen plasma infusions in first 72 h (ml), median (IQR)	0 (0–0)	0 (0–0)	0.6
Sodium bicarbonate administration in first 72 h (ml), median (IQR)	2.2 (1.0–3.8)	2.8 (1.5–4.9)	0.1
Erythrocyte transfusion in first 72 h (ml), median (IQR)	10 (0–15)	11 (0–17.5)	0.7
* Fisher's exact (2-sided)			

### Electrolyte Levels

There were no significant differences in serum sodium and potassium concentrations between the late and early TPN cohort in the first 72 hours after birth (table 3). When adjusted for other sources of sodium, such as infusions of normal saline and sodium bicarbonate administration, no significant differences were found.

**Table 3:** Serum sodium and potassium levels in the first 72 hours after birth of the late and early TPN cohort.

		Late TPN	Early TPN	*P*-Value
		*n* = 70	*n* = 73	
Sodium	Mean (mmol/l), mean (SD)	141.0 (3.7)	141.1 (3.8)	0.91
	Maximum (mmol/l), mean (SD)	147.0 (4.3)	147.1 (4.6)	0.93
	Minimum (mmol/l), mean (SD)	133.7 (4.7)	133.3 (3.4)	0.56
Potassium	Mean (mmol/l), mean (SD)	4.3 (0.5)	4.3 (0.5)	0.92
	Maximum (mmol/l), mean (SD)	5.2 (0.9)	5.3 (0.8)	0.39
	Minimum (mmol/l), mean (SD)	3.6 (0.4)	3.5 (0.4)	0.09

Incidences of hypernatraemia and hyponatraemia were not different between the late TPN cohort and the early TPN cohort (hypernatraemia: 17/70 (24%) vs 23/73 (32%) (*P* = 0.34); hyponatraemia: 17/70 (24%) vs 15/73 (21%) (*P* = 0.59)). Also here, adjustment for additional sodium sources did not influence these findings.

Hyperkalaemia developed in 7/70 (10%) infants in the late TPN cohort, and in 6/73 (8%) infants in the early TPN cohort (*P* = 0.71). Hypokalaemia occurred in 25/70 (36%) vs 39/73 (53%) infants (*P* = 0.03; OR  = 2.1 (95% CI: 1.1–4.0)). There was no significant difference in incidence of hyperkalaemia when an arterial catheter was present in comparison to capillary sample (9/113 (8%) vs 4/17 (19%); *P* = 0.10).

### Fluid Balance

**Figure 1:**
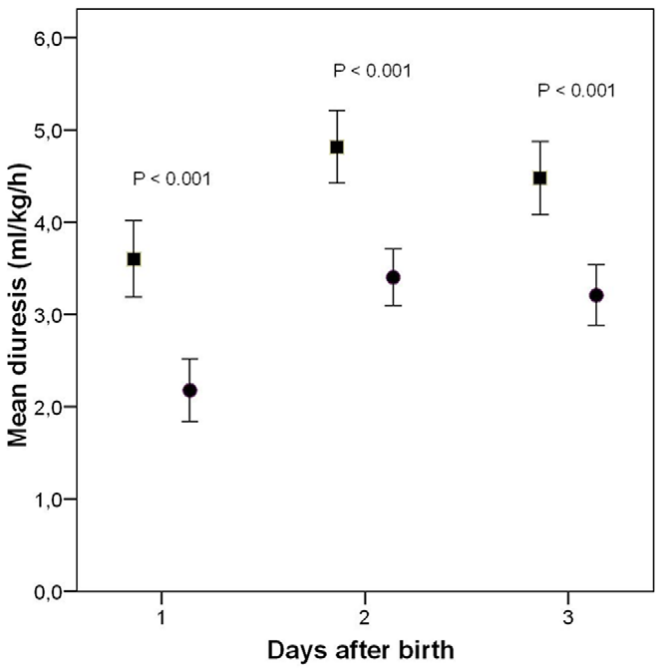
Diuresis in the first days in both cohorts. Diuresis in the first three days after birth in the late (square) and early (circle) TPN cohort. Values are mean; error bars represent 95% CI.

**Figure 2:**
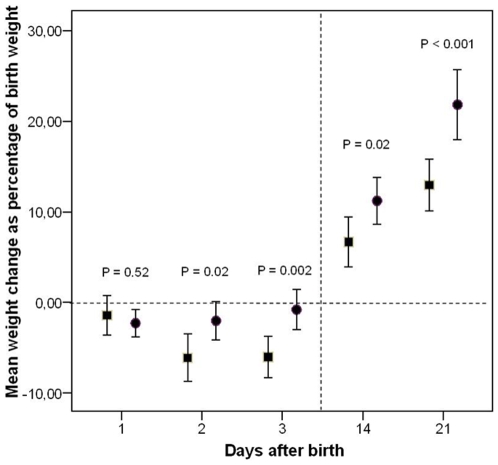
Changes in body weight in both cohorts. Change in body weight as percentage of birth weight on days 1, 2, 3, 14, and 21 after birth for the late (square) and early (circle) TPN cohort. Values are mean; error bars represent 95% CI. Due to hospital transfer or death, valid values for day 14 and 21 are 44/70 (63%) and 37/70 (53%) for the late TPN cohort, and 55/73 (75%) and 49/73 (67%) for the early TPN cohort.

Diuresis in the first three days after birth was significantly lower in the early compared with the late TPN cohort (figure 1). Changes in body weight as percentage of birth weight are presented in figure 2. Loss of body weight in the early TPN cohort was significantly less than in the late TPN cohort on postnatal days 2 and 3. Infants from the early TPN cohort had a significantly higher weight gain on day 14 and 21 than infants from the late TPN cohort (figure 2).

Total daily fluid intakes prescribed in the first three days differ from the intakes recommended in the guidelines. In both cohorts actual fluid intakes were higher than those recommended in the guidelines (table 1 and 4). The mean extra fluid intakes were significantly higher in the early than in the late TPN cohort (late TPN cohort: on day 1, 2, and 3 respectively 2.2 (1.0), 3.9 (1.1), and 6.4 (1.4) ml/kg/day; early TPN cohort: 11.3 (1.3), 12.4 (1.4), and 12.6 (1.5) ml/kg/day; *P*<0.001).

**Table 4:** Actual fluid intake in the first three days after birth of the late and early TPN cohort stratified by birth weight group.

Late TPN cohort			Day 1	Day 2	Day 3
**Birth weight (gr)**					
**<750**	*n = 12*	Fluid intake (ml/kg), mean (SD)	118 (9)	139 (11)	160 (9)
**750–1000**	*n = 40*	Fluid intake (ml/kg), mean (SD)	102 (6)	124 (8)	146 (11)
**1000–1500**	*n = 18*	Fluid intake (ml/kg), mean (SD)	87 (10)	108 (10)	132 (11)
Early TPN cohort					
**Birth weight (gr)**					
**<1000**	*n = 48*	Fluid intake (ml/kg), mean (SD)	88 (9)	110 (11)	130 (13)
**1000–1500**	*n = 25*	Fluid intake (ml/kg), mean (SD)	77 (12)	98 (12)	117 (12)

### Other Clinical Outcomes

There was no difference between the late and early TPN cohort in the need of mechanical ventilation in the first 72 hours (61/70 (87%) vs 54/73 (75%); *NS*), total duration of mechanical ventilation (median days [IQR]: 10 (3–19) vs 11 (3.5–17.5); *NS*), and surfactant treatment (48/70 (70%) vs 48/73 (66%); *NS*). There were no differences in patent ductus arteriosus needing medical or surgical treatment (31/70 (44%) vs 33/73 (45%)), intraventricular hemorrhage (> grade 2) (12/70 (17%) vs 10/73 (14%)), necrotising enterocolitis (≥ stage 2) (1/70 (1%) vs 1/73 (1%)), and cystic periventricular leucomalacia (1/70 (1%) vs 2/73 (3%)). None of the infants in both cohorts had severe retinopathy of the premature (> grade 3).

## Discussion

This study shows that an early administration of TPN and a more restricted fluid intake had a very limited effect on serum electrolytes in very preterm infants in the first three days after birth. Very preterm infants may be able to regulate their electrolyte balance to some degree by modifying renal sodium and potassium excretion in the first days of life, especially during the first 36 hours.[21] Our results underscore the view that hypernatraemia is largely due to a high insensible water loss, rather than an excessive or early intake of sodium.[9], [25] In addition, our findings support that nonoliguric hyperkalaemia does not result from a high or early potassium intake and other causes should be considered. [10], [26], [27]

The incidence of hypernatraemia in our study was comparable to previous reported incidences.[9] Interestingly, in both groups hyperkalaemia occurred less often than reported in other studies.[10], [26], [27] The lower incidence of hyperkalaemia may be explained by the recent increased use of antenatal steroids.[5], [27]

The timing of the introduction of sodium supplementation is a controversial issue in neonatal intensive care medicine and has been addressed by several studies.[11], [13], [21] Two small randomised controlled trials reported lower serum sodium concentrations in preterm infants when sodium intake was restricted from birth.[11], [21] Hartnoll *et al* [13] administrated sodium either on the second day after birth or after a weight loss of 6%, and did not find differences in serum sodium concentrations. We compared initiation of sodium and potassium administration immediately after birth with a moderate sodium and potassium administration started at about 36 hours. However, both cohorts received a substantial sodium intake in the first postnatal days from sources such as sodium bicarbonate, flush fluids (heparin saline), normal saline as volume expander, blood products, and medications.[4] This ‘inadvertent’ sodium load limited the relative difference in total sodium intake in the first days after delivery between both cohorts (table 1).

Our study also shows that the new fluid and nutrition guideline resulted in significant lower urine output and a decreased percentage of body weight loss in the first three days of life. Although the actual fluid intakes were higher than recommended, the decrease in diuresis could reflect the restricted fluid intake of the new guideline. The decreased urine output and weight loss in the first days could suggest that these infants were in better nitrogen and energy balance.[28] However, since this weight loss in the first days is mainly the result of a reduction in the extracellular fluid compartment, a delay in postnatal adaptation (fluid retention) could also be a possible explanation.[4], [6], [7], [12], [15], [21] Early and high sodium administration in preterm infants with a limited ability to excrete sodium[21] will favour persistent expansion of the extracellular fluid compartment and retention of interstitial fluid.[12], [22] This could result in edema formation in the periphery and in the lung, which impedes cardiopulmonary adaptation.[5], [29]

Another explanation for the minimal weight loss in our early TPN cohort in the first postnatal days is that early sodium supplementation led to a gain in intracellular fluid volume and body solids. Low postnatal weight loss was shown to be associated with an increase in intracellular fluid volume, which suggests onset of growth.[5], [12], [30] Also, if energy intake is adequate, early postnatal weight loss can be accompanied by an increase in body solids.[31] The retrospective character of the study can only generate a hypothesis in this, but further research is needed to confirm or invalidate the explanations.

Failure to lose body weight during the physiologic transition as a result of excess fluid or sodium intake has repeatedly been associated with complications of patent ductus arteriosus,[17]–[19] necrotising enterocolitis [19], [20], and respiratory morbidity[13]–[16] in preterm infants. However, other studies did not find any association.[9], [11], [25] This discrepancy might be explained by differences in prescribed fluid intakes and definitions between the studies. The lack of significant differences in the incidence of any of these morbidities, need to taken with caution. The study undertaken was retrospective and not designed to detect such differences. It is difficult to show in a retrospective study if fluid and sodium intake is related to neonatal short term morbidities as more factors could play a role. For this a prospective study is needed.

Interestingly, we found considerable deviations from the prescribed fluid intakes, especially intakes in the ‘restricted’ early TPN cohort were higher than recommended. Apparently, it was not possible to adhere to the restricted fluid intake when an adequate amount of TPN and other necessary infusions had to be given. Possible bias attributable to an adjustment period was minimised, so it seems that adherence to the restricted total fluid intakes in the new guideline is more difficult in practice than anticipated.

The finding of a higher weight gain in the early TPN cohort on day 14 and 21 support the beneficial effect of starting amino acids as soon as possible after birth.[1]–[3] Studies have shown that early administration of the amino acids has positive effects on the nitrogen balance, limiting catabolism, and improves postnatal growth.[1]–[3]

The retrospective character of this study is a major methodological limitation and the results should be interpreted with care. However, other factors which might influence the fluid and electrolyte balance did not change during the course of the study periods. Additional data about sodium balance and body composition would have strengthened our explanations for the similarity in serum electrolytes and the body weight changes. A prospective study design is warranted to definitely assess the effects of a restricted fluid intake and an administration of TPN directly after birth on the rearrangement of body fluids in very preterm infants. Exposure to antenatal steroids might be taken into account to assess their possible positive effects on skin, renal, and pulmonary maturation,[5], [6], [13] thereby reducing IWL, the risk of disturbances in electrolyte levels, and the risk of persistent expansion of the ECF compartment respectively.

In summary, we have shown that an early initiation of TPN immediately after birth combined with a restricted fluid intake in very preterm infants had no influence on serum sodium and potassium levels, but caused a reduced diuresis and lower postnatal weight loss in the first three days of life. Further research is needed to see if a decreased diuresis and loss of body weight in the first days is the result of a delayed postnatal adaptation or better energy balance. The early initiation of TPN also resulted in a higher weight gain after 14 and 21 days after birth, which indicate an early onset of growth. It is possible that the composition of TPN needs to be adjusted to provide for early administration of amino acids, but without sodium and potassium.
